# Resolution of Cerebral Inflammation Following Subarachnoid Hemorrhage

**DOI:** 10.1007/s12028-023-01770-w

**Published:** 2023-06-22

**Authors:** Victor Patsouris, Kinga G. Blecharz-Lang, Melina Nieminen-Kelhä, Ulf C. Schneider, Peter Vajkoczy

**Affiliations:** 1 Institute of Experimental Neurosurgery, Charité – Universitätsmedizin Berlin, Charitéplatz 1, 10117 Berlin, Germany; 2 Department of Neurosurgery, Charité – Universitätsmedizin Berlin, Berlin, Germany; 3 Department of Neurosurgery, Cantonal Hospital of Lucerne, Lucerne, Switzerland; 4 Center for Stroke Research Berlin, Charité – Universitätsmedizin Berlin, Berlin, Germany

**Keywords:** Resolution, Inflammation, Microglia, Neuronal cell death, Cytokines, Subarachnoid hemorrhage

## Abstract

**Background:**

Aneurismal subarachnoid hemorrhage (SAH) is a type of hemorrhagic stroke that, despite improvement through therapeutic interventions, remains a devastating cerebrovascular disorder that has a high mortality rate and causes long-term disability. Cerebral inflammation after SAH is promoted through microglial accumulation and phagocytosis. Furthermore, proinflammatory cytokine release and neuronal cell death play key roles in the development of brain injury. The termination of these inflammation processes and restoration of tissue homeostasis are of utmost importance regarding the possible chronicity of cerebral inflammation and the improvement of the clinical outcome for affected patients post SAH. Thus, we evaluated the inflammatory resolution phase post SAH and considered indications for potential tertiary brain damage in cases of incomplete resolution.

**Methods:**

Subarachnoid hemorrhage was induced through endovascular filament perforation in mice. Animals were killed 1, 7 and 14 days and 1, 2 and 3 months after SAH. Brain cryosections were immunolabeled for ionized calcium-binding adaptor molecule-1 to detect microglia/macrophages. Neuronal nuclei and terminal deoxyuridine triphosphate-nick end labeling staining was used to visualize secondary cell death of neurons. The gene expression of various proinflammatory mediators in brain samples was analyzed by quantitative polymerase chain reaction.

**Results:**

We observed restored tissue homeostasis due to decreased microglial/macrophage accumulation and neuronal cell death 1 month after insult. However, the messenger RNA expression levels of  *interleukin 6*  and  *tumor necrosis factor α* were still elevated at 1 and 2 months post SAH, respectively. The gene expression of *interleukin 1β* reached its maximum on day 1, whereas at later time points, no significant differences between the groups were detected.

**Conclusions:**

By the herein presented molecular and histological data we provide an important indication for an incomplete resolution of inflammation within the brain parenchyma after SAH. Inflammatory resolution and the return to tissue homeostasis represent an important contribution to the disease’s pathology influencing the impact on brain damage and outcome after SAH. Therefore, we consider a novel complementary or even superior therapeutic approach that should be carefully rethought in the management of cerebral inflammation after SAH. An acceleration of the resolution phase at the cellular and molecular levels could be a potential aim in this context.

**Supplementary Information:**

The online version contains supplementary material available at 10.1007/s12028-023-01770-w.

## Introduction

Subarachnoid hemorrhage (SAH) is a type of stroke defined by bleeding into the subarachnoid space, most often due to spontaneous rupture of an intracranial aneurysm or simply because of trauma. Primary brain injury after SAH involves an increase in intracranial pressure, a decrease in cerebral perfusion and blood‒brain barrier (BBB) breakdown that culminates in brain edema [1]. Secondary brain injury following SAH has been mainly associated with ischemic events induced by delayed cerebral vasospasm and cortical depolarization [2, 3]. However, despite achieving a substantial restoration of cerebral vasoconstriction after SAH, large clinical trials failed to develop a clinically effective treatment with an improved outcome [4–6]. These clinical and experimental experiences led to the idea that in addition to vasospasm, other mechanisms may play a decisive role in affecting the general clinical condition, such as cerebral inflammation [7–9]. This conclusion was supported by numerous experimental data. Among others, we have demonstrated in this context how cerebrovascular inflammation triggers intraparenchymal injury [10, 11].

In this work, we focused on tertiary brain damage, which has been defined by others as “injury caused by long-persisting processes following brain insult that worsen outcome, predispose to further injury or prevent repair/regeneration” [12]. Under optimum conditions, after the acute phase of inflammation, direct and indirect mechanisms are implemented to ensure the return to tissue homeostasis. This highly regulated phase called the resolution of inflammation is, according to the current state of research, an active process and not, as it was believed for a long time, exclusively a passive mechanism dependent on cytokine concentration [13–16]. In this phase, the overexpression of proinflammatory mediators should be inhibited, the accumulation of inflammatory cells within the affected tissue attenuated, and furthermore, appropriate mechanisms triggering the recovery from acute inflammation should be activated to constitute a complete recovery of the tissue from inflammation [13–16]. In accordance with the literature, there is strong evidence for a certain resolution program being already set at the beginning of the inflammation phase. Thus, proinflammatory and anti-inflammatory mechanisms seem to affect each other in a direct manner and may have an influence on the extent of resolution. Concerning this highly sensitive interaction, an incomplete resolution of the tissue may therefore increase the risk of chronic inflammation.

In the clinical setting, global brain atrophy or hippocampal volume loss is often observed in addition to long-lasting physical, neuropsychological, and cognitive deficits in patients who have survived SAH [17, 18]. It is therefore reasonable to ask whether chronic inflammatory processes may contribute to these long-lasting impairments or permanent lasting injuries. Regarding these facts, we conducted a long-term observation over 3 months after SAH insult and analyzed histological and molecular parameters in view of the phase called resolution of inflammation. Against this background, we aimed to characterize a potential resolution of cerebral inflammation induced in the well-established model of experimental SAH in mice. Moreover, we aimed to reveal any indications of chronicity accompanying tertiary brain damage driven by an incomplete resolution of inflammation within the affected tissue. Unresolved tertiary brain damage in this context is therefore an important approach to examine the progression and possible chronicity of the inflammatory state occurring after SAH.

## Materials and Methods

### Animals, Experimental Groups, and SAH Mouse Model

Animal experiments were authorized by the corresponding ethics committee and accomplished according to the regulatory guidelines and criteria predetermined by the Landesamt für Gesundheit und Soziales, Berlin, and performed with respect to the European Convention (ETS 123 of 1986). All sections in the manuscript were stated in accordance with Animal Research Reporting in vivo Experiments guidelines. All diagnostic evaluations were performed on the basis of our institutional regulations, and all efforts were made to reduce suffering and total numbers of animals used.

Sixty 12 to 14-week-old male C57BL/6J mice (30–32 g weight) were included in this study and were housed in a 12-h day/night cycle under controlled conditions with free access to food and water. Animals were randomly distributed to one of two groups: sham and SAH. Among the groups, five animals were killed for cryo-tissue immunohistochemical examination and for messenger RNA analysis of coronal sections of brain hemispheres, as indicated in the figures and figure legends.

SAH was induced by the endovascular perforation mouse model, as described elsewhere [10]. Sham-operated mice were operated on identically except that the filament was instantly removed when resistance was noted.

As described before, the induction of experimental SAH was reproducible [10]. Standard hematoxylin/eosin staining excluded territorial infarctions or intracerebral hemorrhage after SAH. Because of the expected occurrence of stroke or intraparenchymal hemorrhage, animals suffering from hemiparesis following the operation were excluded from the experiments [10].

### Mortality

The mortality of animals from all experimental groups was analyzed at 1–3 h, 4–6 h, 6–12 h, 12–24 h, and weekly thereafter. Animal mortality was outlined in the Kaplan‒Meier curve.

### Acute Brain Slice Preparation, Immunohistochemistry, and Image Analysis

Mice in each experimental group were decapitated on days 1, 7, and 14 and months 1, 2, and 3 following SAH. After the skin and skull were removed, the whole brain was detached and washed in ice-cold artificial cerebrospinal fluid (134 mM NaCl, 2.5 mM KCl, 2 mM CaCl_2_, 1.3 mM MgCl_2_, 26 mM NaHCO_3_, 1.25 mM K_2_HPO_4_, and 10 mM glucose).

Immunofluorescence examination of mouse brain specimens was conducted in coronal sections (20 µm), which were prepared with a vibratome VT 1000 S (Leica). Whole mouse brains were cut into two parts for the investigated regions 1.5 mm before and 1.5 mm behind the bregma point (approximately the area of the hippocampus and corpus callosum). Standard hematoxylin/eosin and iron staining were performed to preclude any territorial infarctions or intracerebral hemorrhage at the different time points. Afterward, slides were incubated with primary antibodies at 4 °C for 12 h. Microglia/macrophages were imaged by ionized calcium-binding adaptor molecule-1 (Iba1) immunolabeling (rabbit-anti Iba1, WAKO Pure Chemical Industries, 1:250), and neuronal nuclei (NeuN) (mouse-anti NeuN, Millipore, 1:200) staining was used to identify neurons. Afterward, the following secondary antibodies (Jackson ImmunoResearch Lab) were incubated with the tissue for 1.5 h at room temperature: DyLight651 donkey anti-rabbit (1:200), DyLight488 donkey-anti-rabbit (1:200) and FITC donkey-anti-mouse (1:100). Nuclei were then counterstained with 4′,6-diamidino-2-phenylindole (DAPI)-containing mounting medium (Dianova). For quantitative analysis, images were taken by fluorescence microscopy (Zeiss, Axio Observer Z1, Carl Zeiss, Microimaging GmbH) equipped with a digital camera (AxioCam MRc). Image acquisition of confocal microscopy was obtained with a confocal microscope (TCS SP5, Leica) using a z step of 0.1 µm and a 63 × 1.4 NA oil immersion objective. All images were acquired using LCF AF software (all from Leica). Immunofluorescence areas were divided into 6–10 high power fields allowing for total cell counts per brain section. By using a computer-assisted image analysis program (ImageJ.net), the immunoreactive region was measured. Images were analyzed using ImageTool software, which executes the analysis by converting all immune-labeled elements that fall within a threshold range into black pixels and the rest of the image into white pixels. The software then quantified the total number and percentages of black and white pixels, allowing for statistical analysis of the data.

### Morphology of Microglial/Macrophage Cells

Microglia/macrophages are characterized by strong plasticity and differential morphology. In the healthy brain, most microglial/macrophage cells show a “resting state” with a smaller cytoplasm and poly-branched bodies. However, the branches are very fine and thin [19]. These microglial cells are not actually resting but frequently scan their environment for pathological conditions and regulate neuronal activity [20, 21]. Stepwise deramification of microglial cells with a larger cell body and shorter, thick processes has been observed in different situations of neuroinflammation or after brain injury, indicating an “activated state” [21]. These two differing morphological types additionally produce the spectrum characteristic of in-between phenotypes depending on their activation rate and exact time point of inflammation processes [20]. In the present study, the morphology of microglia/macrophages was described by staining for Iba1 (rabbit-anti Iba1, WAKO Pure Chemical Industries, 1:250). Morphological characterization by using different markers described in previous studies and the number of Iba1-positive cells were used for statistical analysis [22]. Iba1 is expressed either in microglia or macrophages, both of which are indistinguishable due to the expression of most commonly used cell markers in vivo. However, the precision of the staining is limited in injured brain tissue, where peripheral macrophages may infiltrate. Therefore, cells positive for Iba1 were described as microglia/macrophages throughout the article.

### Neuronal Cell Death

Terminal deoxyuridine triphosphate-nickend labeling (TUNEL) (ApopTag Red In Situ Apoptosis Detection Kit, Millipore) was used to detect neuronal cell death in accordance with the manufacturer’s instructions, followed by labeling with NeuN and DAPI. Cells triple-positive for TUNEL, NeuN, and DAPI were rated as neurons undergoing secondary cell death.

### Quantitative Real-Time Polymerase Chain Reaction

RNA isolation from whole brain tissue samples (PureLink RNA Mini Kit, Life Technologies), complementary DNA synthesis (Onestep RT‒PCR Kit, Qiagen) and quantitative real-time polymerase chain reaction (Premix ex Taq Perfect Real Time Kit, Takara) were performed according to the manufacturers’ protocol. For quantitative real-time polymerase chain reaction amplification, we used mouse gene-specific primers (obtained from TIB Molbiol Syntheselabor GmbH) designed using Primer Express Software (listed in Table 1). ABI PRISM 7300 SDS software (Relative quantification study) was used to determine the cycle threshold (CT) for each reaction, and gene expression determined for each gene was normalized to the expression of the endogenous housekeeping gene glyceraldehyde phosphate dehydrogenase. For gene expression analyses the relative quantification method (^∆∆^Ct) was used. The specificity of the PCR products was proven by melting curve analysis.

**Table 1:** Primer sequences used for quantitative real-time PCR

Target	Forward primer 5′–3′	Reverse primer 3′–5′
m_*IL1β*	ATCACTCATTGTGGCTGTGG	CATCTCGGAGCCTGTAGTGC
m_*TNFα*	CACAGCCTTCCTCACAGAGC	GGAGGCAACAAGGTAGAGAGG
m_*IL6*	GAGGATACCACTCCCAACAGACC	AAGTGCATCATCGTTGTTCATACA
m_*GAPDH*	TCTCCTGCGACTTCAACA	TGTAGCCGTATTCATTGTCA

GAPDH, glycerinaldehyd-3-phosphat-dehydrogenase, IL, interleukin, PCR, polymerase chain reaction, TNF, tumor necrosis factor

### Statistical Analysis

GraphPad Prism 6.1 (GraphPad Software) using analysis of variance with pairwise comparison or the Holm‒Sidak method. *P < 0.05, **P < 0.01, ***P < 0.001, and ****P < 0.0001 were used for statistical analysis of data.

## Results

### Mortality

**Fig. 1:**
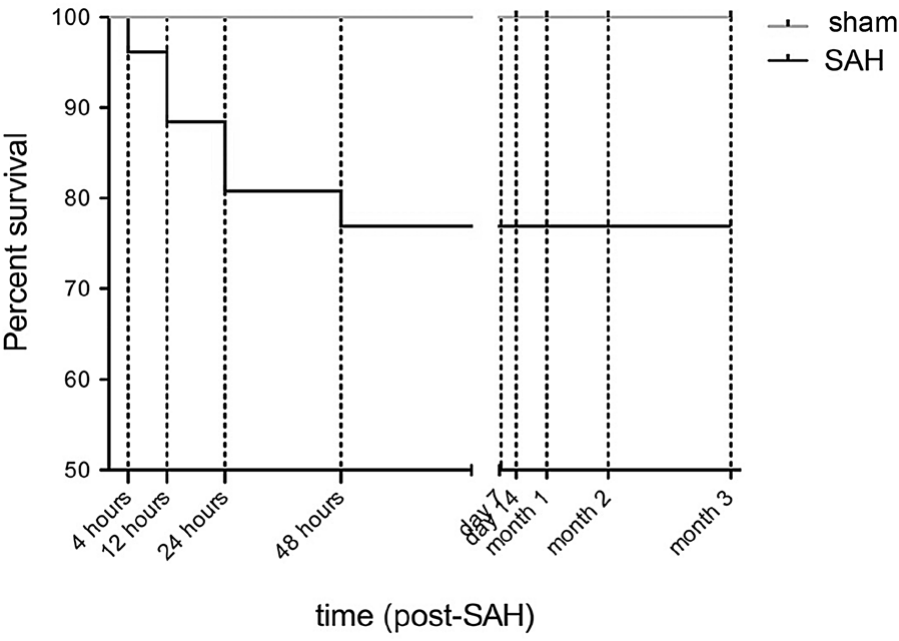
Kaplan–Meier curve displaying posthemorrhagic mortality during a 3-month observation period in the two experimental groups. There was no mortality documented in sham animals. The SAH-operated group exhibited postoperative mortality until day 2 post SAH. SAH, subarachnoid hemorrhage

In the SAH group consisting of 30 animals, 1 animal died within 4 h (3.34%), 2 died 12 h after onset (6.68%), 2 died after 24 h (6.68%), and 1 out of 26 (3.34%) died 48 h post SAH induction. There was no mortality later than 48 h after induction of the bleeding in our study. An overall mortality rate of 20.04% was determined in the SAH group. No mortality was documented in the sham group consisting of 25 animals (Fig. 1).

### Resolution of Microglia/Macrophage Accumulation After Experimental SAH

**Fig. 2:**
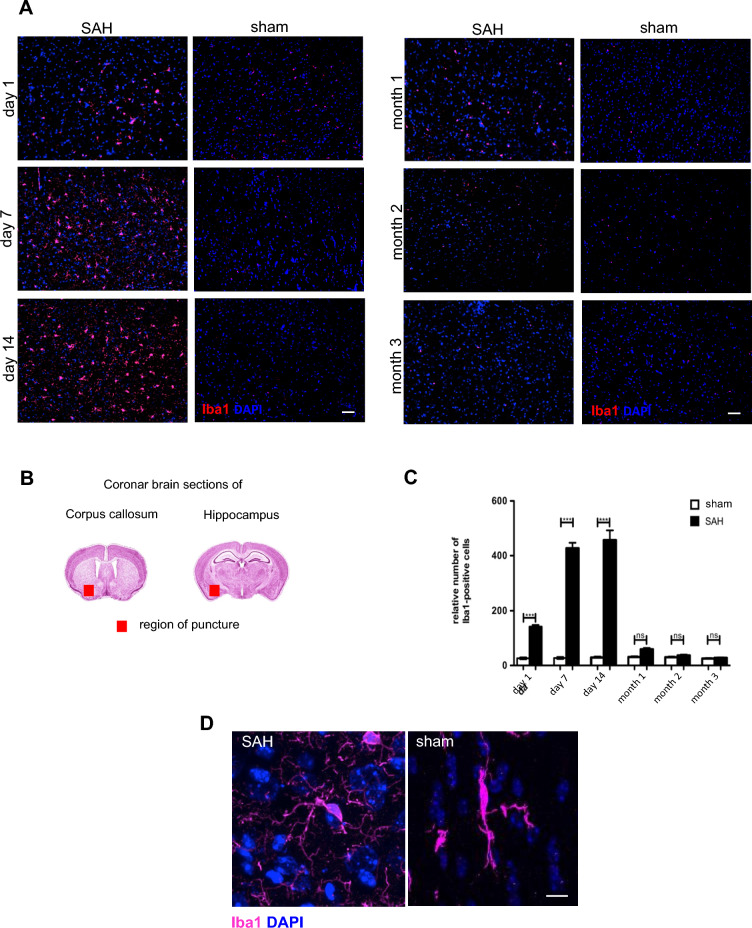
Resolution of microglia/macrophage accumulation after experimental SAH. **a** Coronal brain sections of SAH-operated mice and sham mice were stained for Iba1 (magenta) and DAPI (blue) for different time points (days 1/7/14 and months 1/2/3) to show the time-dependent accumulation of microglia/macrophages and its resolution. Representative brain sections were processed and the number of Iba1-positive cells (red) was counted using ImageJ software. Bar indicates 50 µm. **b** Coronal brain slices were analyzed ipsilateral to the puncture, in the area of the corpus callosum and hippocampus at the base of the brain, as marked by red squares. **c** Microglia/macrophage accumulation was significantly increased in SAH mice compared with sham control until day 14. After month 1, there were no significant differences between the investigated groups, showing a complete resolution of microglia/macrophages on cellular level. Values from graph are means ± SEM, (*n* = 5 animals per group), *P < 0.05, **P < 0.01, ***P < 0.001 and ****P < 0.0001 versus sham respectively, statistical significance determined by one-way ANOVA. **d** Morphology of the “activated” state of a microglia/macrophage on day 7 post SAH in direct comparison to the “resting” phenotype of microglia/macrophages, characterized by a ramified morphology. This “resting” phenotype predominates under sham conditions and after month 1 post SAH, whereas the “activated” phenotype predominates until day 14 post SAH. Bar indicates 10 µm. ANOVA, analysis of variance, DAPI, 4',6-Diamidino-2-phenylindol, Iba1, Ionized calcium-binding adapter molecule 1, SAH, subarachnoid hemorrhage, SEM, standard error of mean  (Color figure online)

To investigate the effect of experimental SAH on the dissemination of microglia/macrophages over a long-term period of up to 3 months, immunohistochemistry of coronal brain sections with an antibody against Iba1 was used in SAH versus sham animals (Fig. 2a). These coronal brain slices were analyzed ipsilateral to the puncture in the area of the corpus callosum and hippocampus, both at the base of the brain (Fig. 2b). Microglia/macrophage accumulation was significantly increased in SAH mice compared with sham controls until day 14 after induction of bleeding, as reflected by the graph in Fig. 2c. This significant time-dependent increase in Iba1-positive cell number was not observed between day 14 and month 1, and after passing month 1, there were no significant differences between the SAH group and the sham-operated mice (Fig. 2a, c). The mean microglia/macrophage density was also much higher in the SAH group than in sham controls over all early time points. A complete resolution of microglia/macrophage accumulation at the cellular level after month 1 was shown for the SAH-operated mice.

Regarding the morphology and activity state of Iba1-positive microglia/macrophage cells observed in this long-term lapse, we examined differences between microglia/macrophages found in sham-operated versus SAH-operated mice. In SAH mice, microglia/macrophages showed an amoeboid and round cell body with long processes, as in a high state of cell activation. Iba1-positive cells in sham and SAH mice after month 1 revealed a low level of activation or inactive state with thin and elongated cell bodies (Fig. 2d). The different morphology and thus activity states of Iba1-positive microglia/macrophages are characterized elsewhere [19].

### Time-Dependent Resolution of Neuronal Cell Death and Neuronal Survival Rate Post SAH

**Fig. 3:**
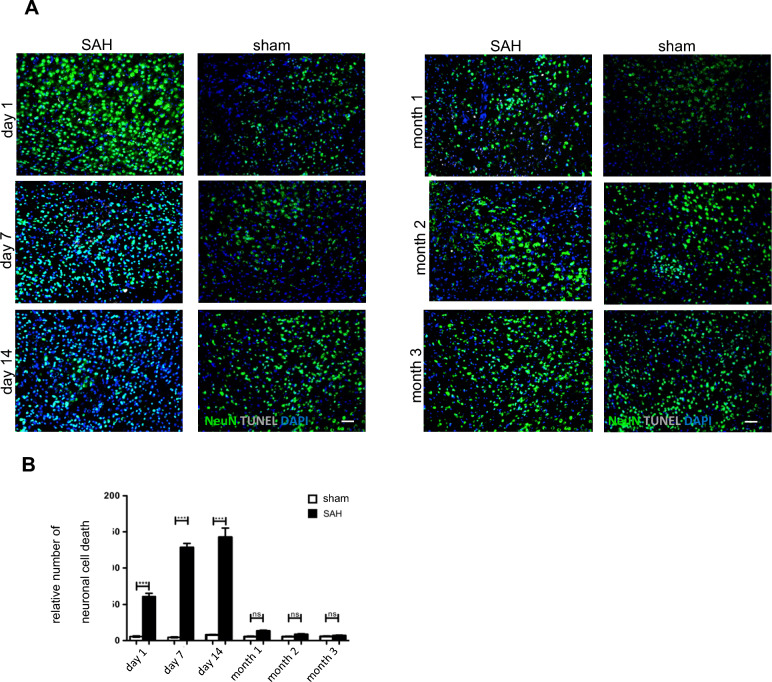
Time-dependent resolution of neuronal cell death and neuronal survival rate following SAH. **a** Animals were killed on days 1, 7 and 14 and on months 1, 2 and 3 following SAH and in the SAH and sham groups. Brain cryosections were immune-labeled for NeuN to visualize neurons and by using TUNEL staining also neuronal cell death was localized. TUNEL-positive (white) NeuN-labeled neuronal cell bodies (green) were counted in regions of interest in all experimental groups and a time course was created. Coronal brain sections were analyzed in the area of corpus callosum and hippocampus, as shown in Fig. 2. Representative images were analyzed using ImageJ software. **b** Neuronal cell death increases significantly in SAH mice compared with sham mice from day 1 on and reaches its peak on day 14. Similar to the microglia/macrophage accumulation, there was no significant difference between the investigated groups after month 1, showing a strong decrease of neuronal cell death between day 14 and month 1. Bar: 50 µm. Values from both graphs are means ± SEM, (*n* = 5 animals per group), *P < 0.05, **P < 0.01, ***P < 0.001, and ****P < 0.0001 versus sham, respectively, statistical significance determined by one-way ANOVA. ANOVA, analysis of variance, DAPI, 4',6-Diamidino-2-phenylindol, Iba1, Ionized calcium-binding adapter molecule 1, NeuN, neuronal nuclei, SAH, subarachnoid hemorrhage, SEM = standard error of mean, TUNEL, Terminal deoxynucleotidyl transferase-nick end labeling (Color figure online)

Microglia/macrophages are known to communicate with neurons and coordinate many neuronal functions [23]. As demonstrated by our experiments with microglia-depleted mice, neuronal cell death following experimental SAH is mediated by the massive accumulation of resident microglial cells [10]. In this context, we also found a more prominent microglia/macrophage–neuronal conjunction either with the cell body or with more than one process of microglial cells in SAH-operated mice, indicating that there is increased interaction between these two cell types in the event of SAH [11]. Regarding neuronal cell death, we used a TUNEL assay and were able to describe an initial increased number of TUNEL-NeuN-DAPI-triple-positive cells in SAH mice until day 14 (Fig. 3a). At month 1, there were no significant differences between the groups, showing complete tissue resolution of neuronal cell death. The significantly lowest neuronal survival rate was observed 14 days post SAH, indicating the highest point of neuronal cell damage. Passing month one, more than 90% of the extant neurons in each SAH group survived. By analyzing the same areas of coronal brain slices we used for microglia/macrophage tissue resolution, a comparison between these two resolution processes was conducted. In particular, by examining the dynamic courses of microglial accumulation (Fig. 2c) and neuronal cell death (Fig. 3b), the association between microglial accumulation and neuronal cell death was clearly recognizable.

### Regulated Gene Expression of Proinflammatory Cytokines in Mouse Brain Homogenates After SAH over an Extended Period of Time

**Fig. 4:**
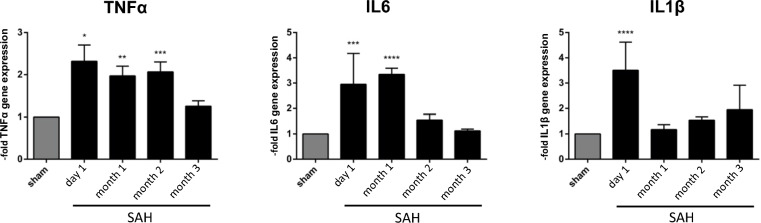
Gene expression of the proinflammatory cytokines *IL6*, *IL1β*, and *TNFα* in brain homogenates after SAH over an extended period of time. Mice were operated and treated as indicated. For each time point following SAH brain tissue was isolated, qPCR was performed, and the results were compared with sham controls. As shown in the graphs, the event of SAH stimulates the gene expression of proinflammatory mediators (here: *TNFα*,  *IL6*, and *IL1β*) over a long period. Only on month 3, the level of gene expression in the SAH group returned to sham levels again, indicating an achieving homeostasis in gene expression for the examined cytokines at the latest considered time point. Values from all graphs are means ± SEM, (*n* = 5 animals per group) ****P < 0.0001, ***P < 0.001, **P < 0.01, and *P < 0.05 versus sham, respectively, statistical significance determined by one-way ANOVA. ANOVA, analysis of variance, IL, interleukin, qPCR, quantitative polymerase chain reaction, SAH, subarachnoid hemorrhage, SEM, standard error of mean, TNF, tumor necrosis factor

Many clinical and experimental studies have noted the important role of different proinflammatory cytokines, such as  *interleukin* (IL) *1β*,  *tumor necrosis factor* (TNF) *α*, and *IL6*, in the context of cerebral inflammation [24]. The effects of experimental SAH on inflammatory cytokine gene expression over a long-term period were determined in whole brain tissue isolated at 1 day and 1, 2, and 3 months after SAH in comparison with the respective sham-operated mice. SAH stimulates the production of proinflammatory mediators by activated microglia/macrophages and other cells of the central nervous system (CNS) [10]. As indicated in Fig. 4, the gene expression of  *IL1β*, *TNFα* and *IL6 *was significantly increased on day 1 after SAH compared with the sham control. In particular, we observed an increase in the gene expression of *IL6* until month 1 post SAH, which then reached sham levels again. Likewise, the gene expression of *TNFα* increased until month 2 and sloped down to sham levels on month 3 after the insult. The gene expression of  *IL1β* reached its maximum on day 1, whereas at later time points, no significant differences between the groups were detected, as reflected by the graph in Fig. 4.

## Discussion

The resolution of cerebral inflammation regarding a possible chronicity of the inflammatory state in the case of incomplete resolution has not yet been considered in the context of SAH, although it seems to play a very important role in the pathophysiology of the disease. Our study demonstrates for the first time a long-term observation of microglial/macrophage accumulation, neuronal cell death and the gene expression of essential proinflammatory cytokines over 3 months after SAH in mice. By evaluating these parameters over an extended period of time, we sought to determine whether the acute inflammatory state occurring after SAH returns to the control level, which is indicative of the resolution of inflammation.

Numerous studies have revealed the importance of resolution in the pathophysiological context of chronic inflammation for different immune-mediated inflammatory diseases [25–27], but also for neurodegenerative diseases such as Parkinson’s disease [28–32]. In accordance with these observations, we were previously able to identify various inflammatory mechanisms, such as an extended release of proinflammatory cytokines, a massive increase of Iba1-positive phagocytic microglia/macrophages (triggered by an intravascular inflammatory response), and increased neuronal cell death following experimental SAH event [10, 11]. Furthermore, we observed that microglia/macrophages first concentrate at the site of the injury, whereupon a spreading inflammation reaction over the rest of the brain occurs, leading to brain damage due to secondary brain injury after SAH [33]. In addition, our group revealed that clinically potential anti-inflammatory attempts preventing microglia/macrophage accumulation or activation are interesting targets for neuroprotective treatment strategies applied after SAH insult [11, 34].

However, inflammation is an essential process in managing many threatening conditions, and when perpetuated and persistent over a long period of time, it contributes to a broad range of chronic diseases [35, 36]. The initiation of an acute inflammatory response is counterbalanced by a fine-tuned and orchestrated resolution process that restores tissue homeostasis and promotes tissue healing to its original structural and functional state. Under pathological conditions, this balance is often disrupted [37]. Although it is not often the primary cause of damage in this context, secondary or tertiary brain damage mediated by an unresolved inflammatory response may constantly disturb the return to homeostasis.

In this work, we confirmed our previous results that microglial/macrophage accumulation is significantly increased after 14 days and is concomitant with the lowest neuronal survival rate after SAH [10, 11]. After month 1, there were no significant differences in microglia/macrophage accumulation observed between the examined groups, suggesting complete resolution with respect to the cell number of these cells (Fig. 2a, c). Regarding their morphology, we saw that the “resting phenotype” with smaller cytoplasm and thin, poly-branched bodies also predominates 1 month after SAH compared with the typical “activated” amoeboid cell morphology with long processes prevailing at every time point until month 1 (Fig. 2d). Moreover, between months 1 and 3 after SAH, more than 90% of the extant neurons survived in each SAH group (Fig. 3b).

SAH stimulates the production of proinflammatory mediators by different cell types of the CNS [10, 38]. In this study, we focused on the gene expression of only three essential cytokines—*IL1β*, *TNFα*, and *IL6*—in the brain. Our results showed that these molecules were significantly upregulated on day 1 after SAH compared with the respective sham control. Although the expression of *IL1β* was already downregulated, the messenger RNA levels of the two others were still increased. Briefly, *IL6* was overexpressed until month 1 post SAH and then reduced to sham levels by month 2. *TNFα* returned to its sham levels even later, 3 months post SAH (Fig. 4). Summarizing, we see that the regulation of *TNFα* and *IL6*, within inflammatory processes takes longer than initially expected. Moreover, searching the literature, we found several studies showing that long-term changes in the proinflammatory cytokines expression, especially in the hippocampus and limbic system, can be correlated with cognitive and emotional dysfunctions in patients after ischemic brain injury [39]. Based on our results, one might speculate that the long-persisting overexpression of essential proinflammatory mediators may correspond with the commonly observed cognitive long-term disabilities accompanying SAH. However, other groups found that modulating neuroinflammation after SAH has a beneficial or no effect on the clinical outcome [40, 41]. Therefore, further specific gene expression analyses of an extended subset of cytokines considering differential brain regions or even specific cell types should be conducted to underpin our early results and correlate them with cognitive dysfunctions following SAH.

Although we did not specify the respective cell types responsible for the overexpression of the proinflammatory markers analyzed herein, we revealed the important role of resolution at the histological and molecular levels after SAH. Mild inflammation is thought to aid in injury repair, and excessive inflammation can aggravate brain edema, mitochondrial dysfunction, disruption of the BBB, and neuronal cell death, all of which cause further impairment of consciousness and are believed to contribute to the poor prognosis of patients with SAH. Such mechanisms might not only apply to CNS insult but also more broadly to physiological conditions in which microglia/macrophages are also known to positively modulate adult hippocampal neurogenesis and where IL6 is also upregulated [42]. Many investigations have documented that besides detrimental effects, IL6 signaling may also play a protective role depending on its activity in different signaling pathways, for example, by counteracting the N-methyl-D-aspartate (NMDA) receptor-mediated excitotoxicity following brain ischemia or the promotion of nerve regeneration [42]. Similar pleiotropic functions and the involvement in tissue remodeling and repair were demonstrated for TNFα and IL1β within the CNS [43, 44]. However, an important question remains whether these cytokines initiate damage, result from damage and promote, halt or repair injury. This may strongly depend on the context, that is, the local concentration, the prevailing environmental milieu, the cellular target, the presence or absence of negative feedback regulators, and the temporal characteristics of the response cascade [43, 44].

IL6 has been confirmed by several authors as a potential biomarker for delayed cerebral ischemia and inflammation following aneurysmal SAH [45–48]. We showed that the concentration of IL6 in the affected whole brain tissue was significantly increased during the acute stage of the disease, reaching a high level on day 1 following SAH, whereas after month 1, no significant difference between SAH and sham controls were observed (as shown in Supplementary Fig. 1). This becomes particularly interesting when looking at CNS-specific microglia, which are cells producing the majority of IL6, and brain capillaries. IL6 expression in brain capillaries is relatively low and is induced in response to inflammatory stimuli, correlating with an increased BBB leakage [38, 49]. Through the damaged BBB, IL6 may enter the bloodstream and trigger a systemic inflammatory response. Given these facts and that IL6 was found to be significantly increased in this time-dependent manner shortly after SAH insult, individual cell and tissue types should be examined for the expression of SAH-induced IL6 regarding the resolution phase. However, subsequent long-term experiments examining changes in the protein expression of a further subset of cytokines should be conducted to point out if the presented preliminary observations support or prevent healing after SAH. Furthermore, the source of the offending cytokines should be identified by cell specific experiments regarding their functional role at certain time points.

Given the current state of research, the resolution of inflammation is a highly regulated, active process and not only a passive burnout reaction, as was once believed [13]. Moreover, it is already encoded at the onset of inflammation and is thereby directly linked to a broad range of inflammatory signaling cascades [13–15]. The main resolution targets for achieving tissue homeostasis include the suppression of further leukocyte influx, tissue clearance of inflammatory cells and the downregulation of proinflammatory cytokines [13–15]. Serhan et al. [13] defined the resolution process at the histological level as the interval from the maximum of cell infiltration to the point when they are lost from the tissue. Numerous scientific works, including ours, have focused on processes arising from the insult itself. Resident microglial cells rather than macrophages recruited from the periphery have been identified to play a triggering role in cerebral inflammation post SAH [10]. In addition, other tissue components seem to play a substantial role in the initiation of neuroinflammatory processes but also create a proinflammatory environment influencing other cell types within the CNS [38]. However, less attention has been given to the essential resolution mechanisms and repair processes of brain tissue to date [16]. Taking these facts and the observations presented here, targeting proresolving factors and mechanisms might be considered a meaningful therapeutic approach in severe inflammatory diseases. Evaluating the best time point for such proresolving involvement would also play an important role in the matter of a possible beneficial influence on the clinical outcome.

In summary, a detailed description of the resolution phase would be very helpful to understand processes involved in brain damage and clinical outcome, as well as its influence on brain repair in the context of secondary brain injury post SAH. There are already differing statements in the literature on this issue [41, 42]. The time point post SAH, at which the inflammatory response or the resolution phase is influenced, as well as the type of cells involved, is decisive for whether neuroinflammatory modulation leads to a protective or harmful effect regarding the resolution phase. The complexity of such processes has to be appreciated by determining differences in the mechanisms of resolution in acute and chronic inflammation as well as inclusion of the tissue-specific environment of the brain after the event of stroke. In addition to these structural changes in the brain tissue, functional read-out parameters through neurobehavioral assessments should also be examined in the future to determine whether structural changes in this context do, indeed, result in a clinically relevant problem [50].

## Conclusions

The key research focus of this study was to present a long-term overview of the time-dependent cerebral processes of inflammation and resolution occurring after experimental SAH and to analyze potential tertiary brain damage due to unresolved inflammation. By evaluating the resolution of inflammation at cellular and histological levels, by estimating the gene expression of three selected cytokines in long-term experiments post SAH, a first step was taken to reveal indications of chronicity driven by incomplete resolution. The intense examination of these issues in the future would provide a better understanding of the long-term mechanisms in the resolution of inflammation and could lead to novel complementary or superior resolution-based therapeutic approaches in the management of cerebral inflammation after SAH.

## Supplementary Information

Below is the link to the electronic supplementary material.

Supplementary file1 (DOCX 68 kb)

## Abbreviations

ANOVA: Analysis of variance
ACSF: Artificial cerebrospinal fluid
BBB: Blood–brain barrier
CNS: Central nervous system
CSF: Cerebrospinal fluid
CT: Cycle threshold
CytC: Cytochrome C
DAPI: 4',6-Diamidino-2-phenylindol
DCI: Delayed cerebral ischemia
GAPDH: Glyceraldehyde phosphate dehydrogenase
HSVTK: Herpes simplex virus thymidine kinase
HPF: High power field
Iba1: Ionized calcium-binding adaptor molecule-1
IL: Interleukin
LPS: Lipopoly-saccharides
MCP-1: Monocyte chemoattractant protein-1
NeuN: Neuronal nuclei
NFκB: Nuclear factor kappa-B
PLX: Pexidartinib
PBS: Phosphate buffered saline
qPCR: Quantitative polymerase chain reaction
SAH: Subarachnoid haemorrhage
SEM: Standard error of mean
TUNEL: Terminal deoxyuridine triphosphate-nick end labelling
TNF: Tumor necrosis factor
