# Supplementary material for: Resolution of Cerebral Inflammation Following Subarachnoid Hemorrhage
Source: Neurocrit Care. 2023 Jun 22;39(1):218–28. doi: 10.1007/s12028-023-01770-w (PMC10499726; doi:10.1007/s12028-023-01770-w)
Supplement: Supplementary file 1 — Supplementary file1 (DOCX 68 kb) [file 12028_2023_1770_MOESM1_ESM.docx]

**Supplementary Material**

**Supplemental Figure 1. Development of the IL6 concentration in brain tissue after day 1, month 1, 2 and 3 following SAH.** In whole brain samples of SAH-operated mice the IL6 protein quantity was examined by using ELISA for different time points and compared to sham mice. The results were summarized in the graph. On day 1 post SAH a significant increase of the IL6 protein concentration could be demonstrated, whereas passing month 1 there were no significant differences between the groups apparent. Values from all graphs are means ± SEM, (n=5 animals per group), **P*<0.05, ***P*<0.01, ****P*<0.001 and *****P*<0.0001 versus sham, respectively, statistical significance determined by One-Way ANOVA.

Abbreviations: ANOVA = analysis of variance; ELISA = enzyme-linked immunosorbent assay; IL = interleukin; SAH = subarachnoid hemorrhage; SEM = standard error of mean

**
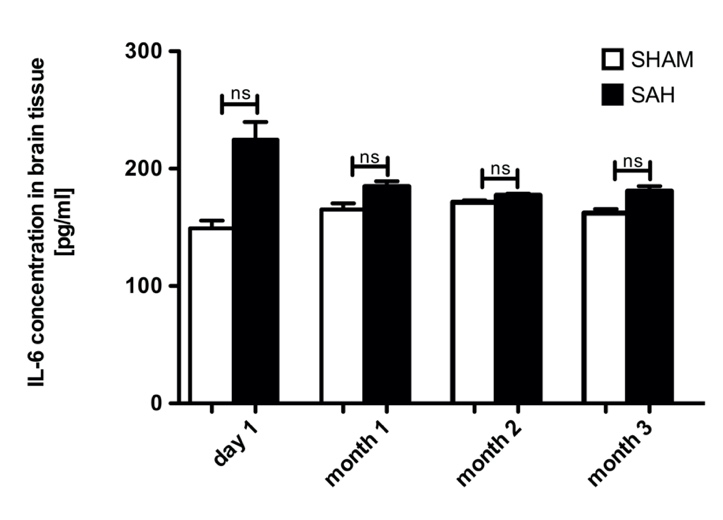
**

*******

**IL6 concentration in brain tissue (pg/ml)**

sham
